# Knocking-down of the Prokineticin receptor 2 affects reveals its complex role in the regulation of the hypothalamus-pituitary-gonadal axis in the zebrafish model

**DOI:** 10.1038/s41598-020-64077-2

**Published:** 2020-05-06

**Authors:** Ivan Bassi, Francesca Luzzani, Federica Marelli, Valeria Vezzoli, Ludovica Cotellessa, David A. Prober, Luca Persani, Yoav Gothilf, Marco Bonomi

**Affiliations:** 1 IRCCS Istituto Auxologico Italiano, Division of Endocrine and Metabolic Diseases & Lab. of Endocrine and Metabolic Research, Milan, Italy; 2 Department of Clinical Sciences and Community Health, University of Milan, Milan, Italy; 3 Division of Biology and Biological Engineering, California Institute of Technology, Pasadena, CA USA; 4 Department of Neurobiology, The George S. Wise Faculty of Life Sciences and Sagol School of Neuroscience, Tel-Aviv University, Tel-Aviv, Israel

**Keywords:** Neurogenesis, Endocrinology

## Abstract

Prokineticin receptors (PROKR1 and PROKR2) are G protein-coupled receptors which control human central and peripheral reproductive processes. Importantly, allelic variants of *PROKR2* in humans are associated with altered migration of GnRH neurons, resulting in congenital hypogonadotropic hypogonadism (CHH), a heterogeneous disease characterized by delayed/absent puberty and/or infertility. Although this association is established in humans, murine models failed to fully recapitulate the reproductive and olfactory phenotypes observed in patients harboring *PROKR2* mutations. Here, taking advantage of zebrafish model we investigated the role of *prokr1b* (ortholog of human *PROKR2*) during early stages of GnRH neuronal migration. Real-Time PCR and whole mount *in situ* hybridization assays indicate that *prokr1b* spatial-temporal expression is consistent with *gnrh3*. Moreover, knockdown and knockout of *prokr1b* altered the correct development of GnRH3 fibers, a phenotype that is rescued by injection of *prokr1b* mRNA. These results suggest that *prokr1b* regulates the development of the GnRH3 system in zebrafish. Analysis of gonads development and mating experiments indicate that *prokr1b* is not required for fertility in zebrafish, although its loss determine changes also at the testis level. Altogether, our results support the thesis of a divergent evolution in the control of vertebrate reproduction and provide a useful *in vivo* model for deciphering the mechanisms underlying the effect of *PROKR2* allelic variants on CHH.

## Introduction

The hypothalamus-pituitary-gonadal (HPG) axis controls reproduction in vertebrates^1–5^ through the pulsatile release of gonadotropin-releasing hormone (GnRH) hormone by GnRH neurons. During development, these neurons differentiate from neural crest cells and ectodermal progenitors in a niche at the border between the respiratory epithelium and the vomeronasal/olfactory epithelium. From these regions these neurons migrate caudally to reach the medio-basal hypothalamus, where they complete their differentiation^6^. Several studies in different animal models have shown that specific evolutionarily conserved genes regulate this migratory process and the functions of these neurons. The role of these genes in human fertility is supported by the existence of variants in these genes associated with congenital hypogonadotropic hypogonadism (CHH)^7^, a rare and clinically heterogeneous disorder characterized by abnormal pubertal development and/or infertility, a normal (nCHH) or defective sense of smell (Kallmann syndrome, KS) and other reproductive and non-reproductive anomaliess^8–10^. More than 25 genes have been associated with CHH, although variants in these genes account only for 40–50% of reported cases. Several evidences indicate that CHH is a complex genetic disease characterized by variable expressivity and penetrance of the associated genetic defects, which can be partially explained by an oligogenicinheritance model^9,11^. Among the genes involved in CHH, prokineticin receptor 2 (*PROKR2)* has an important role in GnRH neuron migration. PROKR2, as a member of the GPCR family, has an extracellular amino-terminal end, an intracellular carboxy-terminal domain and a central core formed by seven transmembrane α-helical segments (TM1–TM7)^12^. Prokineticins have been previously demonstrated to be involved in several physiological functions in neurogenesis, regulation of circadian rhythms, metabolism, angiogenesis, pain perception, muscle contractility, hematopoiesis, immune response, thermoregulation and energy expenditure^13–15^. Matsumoto and colleagues in 2006 reported the first observation that *Prokr2* knock-out mice display hypoplastic gonads and olfactory structures, a phenotype reminiscent of KS, to link this gene to CHH^16^. Furthermore, *Prokr2* knock-out mice show decreased plasma levels of testosterone and follicle-stimulating hormone, but not luteinizing hormone. In humans, genetic screening of CHH cohorts has revealed mutations in *PROKR2* in KS and nCHH patients, but mostly in the heterozygous state^17–20^. Moreover, studies on transfected cells demonstrated that the missense *PROKR2* variants observed in KS and nCHH patients have a deleterious effect on *PROKR2* signaling^18,20,21^ although such variants are also present in apparently unaffected individuals^22–24^. Thus, despite several *in vitro* and *in vivo* studies, the role of Prokr2 in GnRH neuron function and in CHH pathogenesis remains incompletely understood. Here, we take advantage of the zebrafish to generate an *in vivo* model to investigate *PROKR2* function during GnRH neuron ontogeny. Using both transient knockdown and germline knockout of *prokr1b*, the zebrafish ortholog of human *PROKR2*, we find that *prokr1b* has an important role in the migration of GnRH axons, but not for fertility, in this animal model.

## Results

**Figure 1:**
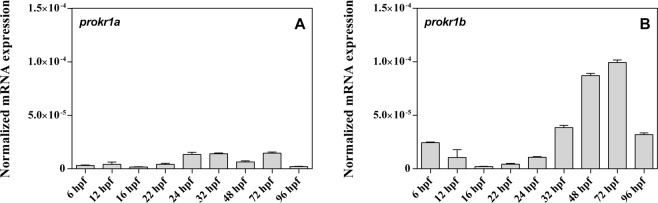
Real-time-PCR experiments show that during GnRH3 neuron development *prokr1a* (**A**) is less expressed compared to *prokr1b* (**B**). (gene were normalized using *eef1a* as housekeeping gene. For each developmental stage n = 20).

Mammals possess two prokineticin receptors named *PROKR1* and *PROKR2*^25^. The zebrafish genome contains two *prokr* paralogs named *prokr1a* and *prokr1b* (or *prokr1l*), and previous evidence suggests that *prokr1a* and *prokr1b* correspond to mammalian *PROKR1* and *PROKR2*, respectively^26^. To determine which of these genes may be involved in GnRH neuronal migration, we evaluated their expression during zebrafish embryo development. Real-Time PCR analysis on RNA extracts from whole embryos revealed that *prokr1b* is expressed at higher levels compared to *prokr1a* and exhibits an increase from 24 hours post-fertilization (hpf) to 72 hpf (Fig. 1A,B).

**Figure 2:**
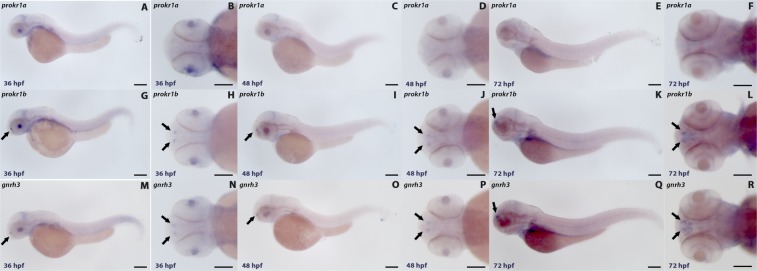
Whole-mount *in situ* hybridization with *prokr1a* probe (**A–F**) did not display any signal in the head of zebrafish embryos. In contrast, *prokr1b* (**G–L**) is expressed in cells near the olfactory region at 36 hpf (**G**,**H**), 48 hpf **(I,J**) and 72 hpf (**K,L**). That is similar to *gnrh3* expression at these times (**M–R**). Panels A,C,E,G,I,K,M,O and Q show lateral views. Panels B,D,F,H,J,L,N,P and R show dorsal views of zebrafish embryos. Scale bar = 50 µm. Image taken using Leica Application Suite software (LAS version 4.7.0; https://www.leica-microsystems.com/products/microscope-software/p/leica-application-suite/).

Whole-mount *in situ* hybridization (WISH) performed at the same developmental stages (Fig. 2) revealed that *prokr1b*, but not *prokr1a*, is expressed in cells adjacent to the olfactory placodes (Fig. 2G-L), similar to the pattern observed for *gnrh3* (Fig. 2M-R). Given the role of *PROKR2* in the migration of GnRH neurons in humans, these results suggest that *prokr1b* may similarly be involved in GnRH3 neuron development in zebrafish.

**Figure 3:**
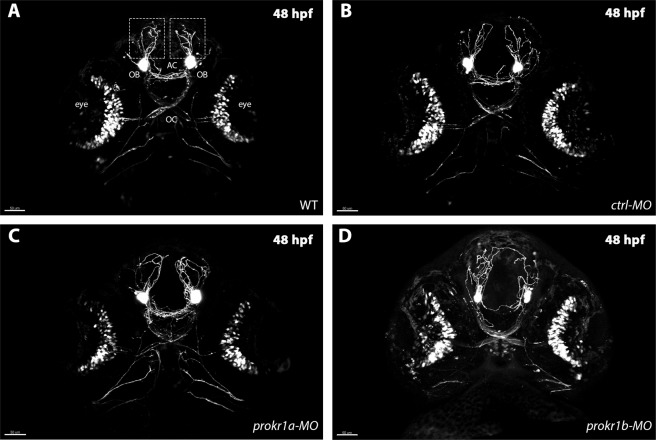
Injection of *ctrl-MO* (**B**) or *prokr1a-MO* (**C**) does not affect GnRH3 neuron fibers, as they look comparable to uninjected WT (**A**) embryos. In contrast, knockdown of *prokr1b* (**D**) causes alteration in axons migration of the rostral part (dotted square in Fig A) and at the level of the anterior commissure (AC) of the embryos. OB = olfactory bulbs, OC = optic chiasm, AC = anterior commissure. Scale bar=50 µm.

Next we used morpholino anti-sense oligonucleotides (MOs) to knock-down expression of *prokr1a* or *prokr1b* in *tg (gnrh3:EGFP)* embryos, which express EGFP in GnRH3 cells^27^ (Supplementary Fig. S1A–C). Images collected at 48 hpf (Fig. 3) revealed that *prokr1a* knock-down (Fig. 3C) did not affect the development of GnRH3 fibers that appeared similar to those of uninjected wild-type animals (WT, Fig. 3A) or injected with a control MO(*ctrl-MO)* (Fig. 3B). In contrast, knock-down of *prokr1b* led to evident alterations in the architecture of the GnRH3 network (Fig. 3D). In these embryos, GnRH3 fibers appeared disorganized, especially at the level of the anterior commissure (AC) and in the anterior fibers (dotted square in Fig. 3D; Supplementary Fig. S1D). These results suggest that *prokr1b* is required for normal GnRH neuron development in zebrafish.

**Figure 4:**
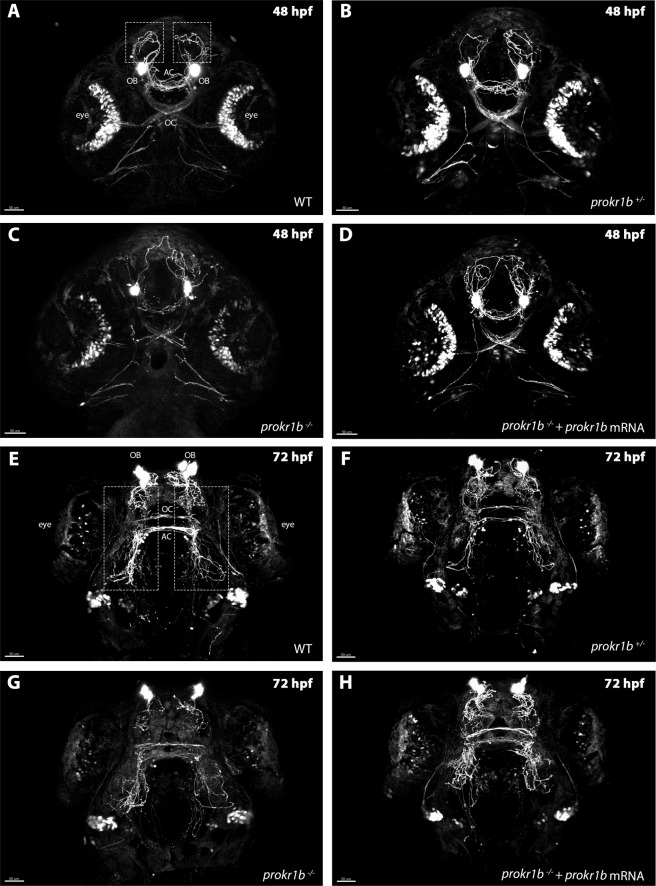
*prokr1b* homozygous mutant zebrafish (*tg(gnrh3:EGFP);prokr1b*^−/−^ (**C,G**) exhibit misrouting of rostral (dotted square) and optic chiasm (OC) fibers. These defects are not present in WT (**A,E**) and *prokr1b* heterozygous mutants (*tg(gnrh3:EGFP);prokr1b*^*+/-*^ (**B,F**). The *prokr1b* mutant phenotype is rescued by injection with WT *prokr1b* mRNA (**D,H**). OB = olfactory bulbs, OC = optic chiasm, AC = anterior commissure. Scale bar = 50 µm.

To confirm the defects observed for *prokr1b* knock-down using MOs, a technique that is prone to non-specific artefacts, we next analyzed GnRH3 neuron development in animals containing a germline mutation in *prokr1b*^26^. To do so, we compared EGFP-expressing GnRH3 neuron fibers in *prokr1b* homozygous mutant, heterozygous mutant, and homozygous WT siblings at 48 hpf and 72 hpf. No differences were observed between *prokr1b*^+*/−*^ and WT siblings at these two time points (Fig. 4A,B,E,F), while *prokr1b*^−/−^ embryos (Fig. 4C,G) showed defects in GnRH3 neuron fibers in the same anatomical region as in the knockdown experiments (Fig. 3D). This phenotype appears to be specific to the absence of *prokr1b*, as injection of WT *prokr1b*, but not *prokr1a* mRNA into *prokr1b*^−/−^ animals at the 1-cell stage rescued the phenotype (Fig. 4D–H; Supplementary Fig. S2E,F).

**Figure 5:**
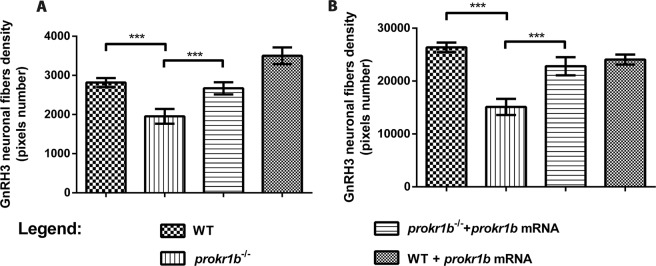
Quantification of GnRH3 fiber network confirms that *prokr1b*^−/−^ embryos have reduced GnRH3 neuron projections, and that this phenotype is rescued by injection of *prokr1b* mRNA when analyzed at 48 hpf (**A**) or 72 hpf (**B**). For each group n = 10 animals. ns: not significant, ***p < 0.01.

These observations were supported by quantitative analysis of GnRH3 neuron fibers(Fig. 5A,B), providing an evidence for a role of *prokr1b* in GnRH neuron development in zebrafish.

**Figure 6:**
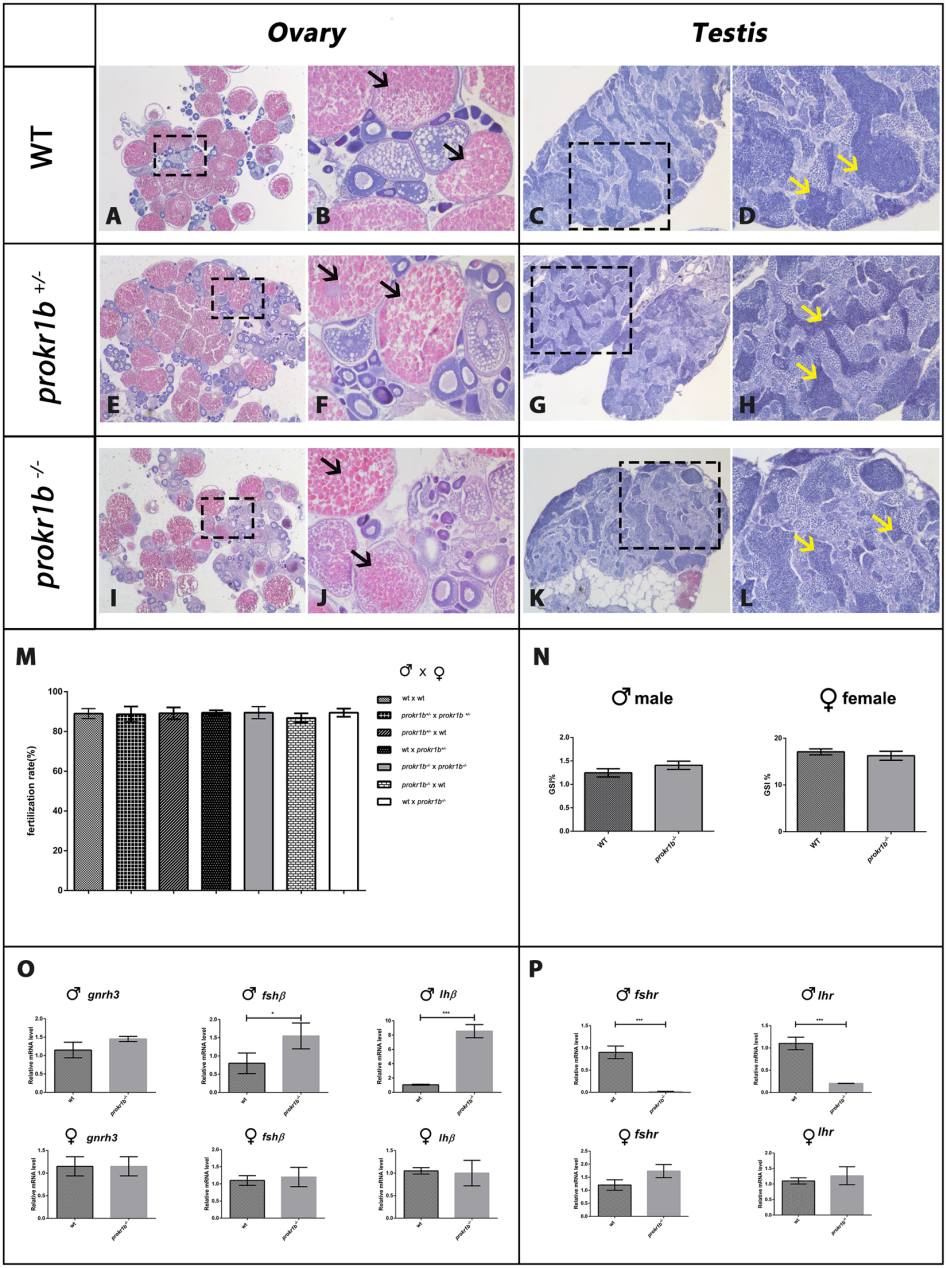
Histological sections conducted on 3 months of age fish showed no defects in the structure of gonads and testis between WT (**A,C**), heterozygous (**E,G**) and homozygous mutant (**I,K**). Sections zoomrevealed fully mature oocytes (black arrows in **B,F,J**) and fully mature spermatozoa (yellow arrows in **D,H,L**) in WT,*prokr1b*^+/−^ and *prokr1b*^−/−^ animals. Comparison of reproductive outputs (**M**) and GSI index (**N**) revealed no differences between WT, heterozygous and homozygous mutants. Real-Time PCR conducted on brain (**O**) and gonads (**P**) of WT and mutant fish showed a different expression of *lhβ*, *fshβ*, *lhr* and *fshr* in *prokr1b*^−/−^ males. Image taken using Leica Application Suite software (LAS version 4.7.0; URL: https://www.leica-microsystems.com/products/microscope-software/p/leica-application-suite/).

In order to establish whether *prokr1b* is required for the development and function of the reproductive system in zebrafish, as it is in humans and mice, we compared the reproductive organs and fecundity of *prokr1b*^−/−^, *prokr1*^+*/*-^ and *prokr1b*^+/+^ siblings. Histological sections of testes and ovaries of 3-months-old male and female animals did not reveal obvious differences among the three genotypes, suggesting that *prokr1b* is not required for gonadal maturation in both sexes and, by consequence, for puberty in zebrafish (Fig. 6A-L). No differences were also observed among the three genotypes in the number of fertile eggs generated (Fig. 6M), neither in the GSI index of mutated male or female compared to WT indicating that *prokr1b* is not fundamental for fertility in zebrafish (Fig. 6N). Nevertheless, Real-Time qPCR conducted on tissue of adult fish, revealed for mutated males higher expression of *lhβ* and *fshβ* in the brain (Fig. 6O) together with a strong decrease of the gonadotropic receptors *lhr* and *fshr* in the gonads (Fig. 6P).

## Discussion

Several recent studies have demonstrated a remarkable evolutionary conservation of the developmental migration of GnRH neurons and of several genes involved in GnRH ontogeny^28–31^. In zebrafish, like in mammals, GnRH secreting neurons starts their development at 24 hpf from cells located in the olfactory epithelium that send dorsal extensions that ultimately innervate the hypothalamus and pituitary. An important gene involved in the development of the GnRH system in humans is *PROKR2*. The zebrafish genome contains two *prokineticin receptor* paralogues, *prokr1a* and *prokr1b*^26^. WISH analyses reveal that *prokr1b* expressionstarts in the brain at 24 hpf close to the olfactory bulbs and appears similar to that of *gnrh3* at 48 and 72 hpf. Accordingly, Real-Time PCR data show that during this time window *prokr1b* expression increases and then drastically decreases at 96 hpf. At 72 hpf, the development of GnRH3 fibers is complete and is followed by the migration of GnRH3 somata from the olfactory region to the hypothalamus^27^. Consistent with the possibility that zebrafish *prokr1b* is an ortholog of human *PROKR2*, knockdown of *prokr1b*, but not *prokr1a*, affected the formation of rostral GnRH3 fibers, similar to humans with *PROKR2* mutations. Importantly, similar defects were also present in homozygous *prokr1b* mutant embryos at 48 hpf, and this phenotype was rescued by injection of WT *prokr1b* mRNA. Taken together, these results demonstrate that *prokr1b* is important for the correct migration of GnRH3 neuron fibers.

Although *prokr1b* appears to be the zebrafish ortholog of human and murine *PROKR2*, the zebrafish *prokr1b* mutant phenotype does not fully recapitulate the clinical features of CHH. Indeed, *prokr1b* mutation does not affect gonadal maturation or fertility in zebrafish, as demonstrated by fecundity testing and histological analysis of testis and ovaries at 3 months of age. Moreover, dorsoventral projections of GnRH3 neurons, despite reduced, are present in zebrafish *prokr1b* mutants at 72 hpf, in contrast to murine *Prokr2* mutants in which there is an early arrest in GnRH neuronal migration^16^. Two recent studies conducted in zebrafish have highlighted differences in the role of the GnRH system during puberty and fertility. Liu and colleagues showed that triple mutants lacking *gnrh3* and the 2 kisspeptin ligands undergo normal puberty and gonad maturation^32^. These results are surprising, because GnRH3 has been considered the most important stimulator of gonadotropin release in fish and its expression, together with *kiss1* and *kiss2*, have been found to be higher during puberty and gonadal maturation in zebrafish^33^. Moreover Marvel and colleagues demonstrated that zebrafish *gnrh2*/*gnrh3* double mutants show normal fertility, demonstrating that neither GnRH2, nor Kiss1 and Kiss2, compensate for loss of GnRH3 in zebrafish^34^. Nevertheless, comparison between WT and double or triple mutants revealed in both studies different expression patterns of neuropeptides known to be important in mammal control of reproduction, such as *tachykinin 3*, *secretogranin II* and *neuropeptide Y*. These results suggest that, in contrast with mammals, multiple factors act in parallel with GnRH to stimulate the reproductive axis in zebrafish^32,34,35^. Our results in the knockout male fish might further confirm this hypothesis. Indeed, we reported a *lhr* and *fshr* lower expression in the testis that could firstly indicate a role of *prokr1b* in this organ similar to what already observed in mice, where absence of *Prokr2* lead to a variable degree of compromised vasculature, even in the absence of evident structural gonadal modification^36^. Moreover, this could also be related to primary testes defect that has been described, in association to those in the hypothalamus and pituitary, also in human male with CHH (Sykiotis *et al*. JCEM 2010). Secondly, this reduced receptor expression might lead to a relative resistance to Lh and Fsh action which, in turn, might activates, through the negative feedback mechanism, the central compartment of the HPG axis. Higher *lhβ* and *fshβ* expression levels in our male knockout fish, seem to confirm this stimulation, nevertheless they are not consequence of level modification in *gnrh3* expression levels. Thus, other factors from GnRH3 system might be implicated in the stimulation of the pituitary as previously suggested^32,34,35^.

In conclusion, even if mechanisms controlling the HPG and, by consequence, fertility have slightly diverged along evolution, these studies together demonstrate that genes regulating GnRH ontogeny present a certain degree of conservation among humans, mice and zebrafish^37,38^. Indeed, despite the variable phenotypic features, the *Tg(gnrh3:EGFP);prokr1b*^*ct814/ct814*^ line presented here suggests that *prokr1b* is the orthologue of human *PROKR2*, and demonstrates that its loss affects the development of GnRH neuronal fibers in zebrafish, asin humans, but also the expression of the *lhr* and *fshr* at the testes level, thus indicating a complex implication of the prokineticin pathway in the HPG functionality. Moreover, this mutant lineis a useful *in vivo* tool that, combined with mutant lines for other GnRH related genes, could contribute to our understanding of the development of the GnRH system and the complex mechanisms underlying CHH and related diseases.

## Methods

### Zebrafish lines and maintenance

Zebrafish (*Daniorerio*) embryos obtained from natural spawning were raised and maintained according to established techniques^39^. All experiments with live animals were performed at the University of Milan. All experimental protocols and methods were carried out in accordance with relevant guidelines and regulations of Good Animal Practice approved by the institutional and licensing committee IACUC (Institutional Animal Care and Use Committee) and University of Milan by the Italian Decree of March 4th, 2014, n.26. Embryos were staged according to morphological criteria^40^. Beginning from 24 hpf, embryos were cultured in fish water containing 0.003% PTU (1-phenyl-2-thiourea; Sigma–Aldrich, Saint Louis, MO) to prevent pigmentation and 0.01% methylene blue to prevent fungal growth^39^. Wild-type (WT) zebrafish of the AB strain were obtained from the Wilson lab (University College London, London, United Kingdom). The *tg (gnrh3:EGFP)*^27^ and *prokr1b*^ct814/ct814 26^ zebrafish lines have been previously described.

### Real-Time PCR

Reverse Transcription-Polymerase Chain Reaction (RT-PCR) was performed on total RNA prepared from 20 zebrafish oocytes and embryos for each different developmental stages using the Total RNA Isolation Kit (Ambion, Thermo Fisher, Waltham MA) or the RNAgents Total RNA Isolation System (Promega, Madison, WI), treated with DNase I RNase free (Roche, Basel, Switzerland) to avoid possible contamination from genomic DNA. RNA concentrations and quality were determined using a NanoDrop ND-1000 spectrophotometer (NanoDrop Technologies Inc., Wilmington, USA). Total RNA (1 ug) was reverse transcribed to produce cDNA using Superscript III reverse transcriptase (Invitrogen) primed with random hexamers, as described previously^41^. In all cases, a reverse transcriptase negative control was used to test for genomic DNA contamination. The primers used for quantitative Real-Time PCRare listed in the Supplementary Table S1.

### *In situ* hybridization

Whole-mount *in situ* hybridization (WISH) was performed as described^42^. PCR products were cloned into the pGEM-T Easy vector (Promega, Table S2). The cDNA-containing plasmids were linearized and transcribed with T7 and SP6 RNA polymerase (Roche) for antisense and sense riboprobe synthesis. Images of stained embryos were taken with a Leica MZFLIII epifluorescence stereomicroscope equipped with a DFC 480-R2 digital camera.

### Knockdown experiments

We tested one antisense morpholino oligonucleotide (MO) each for *prork1a* and *prokr1b* (Supplementary Table S3). Both were splice-blocking MOs synthesised by Gene Tools LLC (Oregon, USA). Morpholinos were dissolved in Danieau’s solution (58 mMNaCl; 0,7 mMKCl; 0,4 mM MgSO_4_. H_2_O; 0,6 mMCa(NO_3_)_2_; 5 mMHepes pH 7.2) at 2 mM and stored at −80 °C. Embryos were microinjected at the 1–4 cell stage with rhodamine dextran (Molecular Probes) co-injected as a tracer. As a control for non-specific effects, a standard control morpholino (*ctrl-*MO) was injected, which targets the human *β-globin* gene. Morpholinos were tested for efficacy and toxicity by injecting different doses in *tg(gnrh3:EGFP)* embryos and evaluating them for morphological defects (Supplementary Fig. S1A-C). After injection, embryos were raised in fish water at 28 °C and observed until the developmental stage of interest. Embryos that were to be imaged after 24 hpf were treated with PTU. For imaging, embryos were anaesthetized using tricaine (ethyl 3-aminobenzoate methanesulfonate salt, Sigma; 25x stock = 0.08 g in 20 ml of distilled H_2_O) in fish water. Injected embryos (morphants) were embedded at 48 hpf in UltraPure Low Melting Point Agarose (Thermo Fisher Scientific) and photographed using a confocal laser scanning microscope (Nikon C2) with a 20x objective.

### Generation of *tg(gnrh3:EGFP)*; *prokr1b*^ct814/ct814^ line and rescue experiments

We crossed *tg(gnrh3:EGFP)*^27^ animals to *prokr1b*^ct814/ct814^ animals^26^ to generate the *tg(gnrh3:EGFP)*; *prokr1b*^ct814/ct814^ line. Fin clipping was performed to isolate genetic material from individual fish for genotyping accordingly to what previous published in Chen and colleague^26^ (Table S4). The *prokr1b*^ct814/ct814^ fish contain a 1 bp deletion (nucleotide 12 of the open reading frame: 5’-C-3’), which results in a change in reading frame after amino acid 4 and a premature stop codon after amino acid 13 compared to 396 amino acids for the wild-type (WT) protein. Rescue experiments were performed by injecting 400 pg *prokr1b* mRNA diluted in the Danieau’s solution into 1-cell stage embryos.

### Live-imaging of GnRH3 fibers in *prokr1b* KO embryos

To assess the role of *prokr1b* during GnRH3 fiber development, *prokr1b* KO animals were embedded at 48 or 72 hpf in UltraPure Low Melting Point Agarose (Thermo Fisher Scientific) and analysed using a confocal laser scanning microscope (Nikon C2+) with a 20x objective. GnRH3 fiber structure was assessed and 3D reconstructed using Fiji^43^. Due to the complexity of GnRH3 fibers, a specific region of interest (ROI) was selected and analyzed at each developmental stage (Supplementary Fig. S2), with background fluorescence subtracted from each image (Supplementary Fig. S2B–D). The number of green pixels within each ROI was used as a proxy for the amount of GnRH3 fibers.

### Gonads histology, fecundity/fertilization ratesand GSI

For histological analysis, gonads from 3-months-old fish were fixed in 4% paraformaldehyde (PFA), dehydrated, wax-embedded, cut into 8 µm sections using a microtome (Leitz 1516), and stained with eosin. Samples were imaged using a Leica DM6000 B microscope equipped with a Leica 480 digital camera using the Leica Application Suite (LAS version 4.7.0). The assessment of fecundity (number of eggs released) and fertilization rate (fraction of eggs that developed into an embryo), WT and mutant females and males at 3 months-old were paired in a spawning tray. After one hour, eggs were collected in 30% Danieau’s solution and counted. The number of fertilized and unfertilized eggs was discerned using a dissecting microscope at 6 hpf. Twelve-months-old male and female fish were then dissected to collect ovaries and testicles for gonadosomatic index (GSI) measurement and Real-time PCR. The GSI was calculated according to the formula (organosomatic index = organ weight × 100/body weight)^44^.

### Statistical analysis

Statistical analyses in Fig. 5 was performed using one-way ANOVA with Dunnett’s post-hoc test using GraphPad PRISM version 6.0 (GraphPad, San Diego, CA). In the graphs, *P < 0.05, **P < 0.01, ***P < 0.001.

## Supplementary information

Supplementary information.

Supplementary information2.

Supplementary information3.

Supplementary information4.

Supplementary information5.
