# Publisher Correction: Prokineticin receptor 2 affects GnRH3 neuron ontogeny but not fertility in zebrafish

**DOI:** 10.1038/s41598-020-65551-7

**Published:** 2020-05-15

**Authors:** Ivan Bassi, Francesca Luzzani, Federica Marelli, Valeria Vezzoli, Ludovica Cotellessa, David A. Prober, Luca Persani, Yoav Gothilf, Marco Bonomi

**Affiliations:** 1 IRCCS Istituto Auxologico Italiano, Division of Endocrine and Metabolic Diseases & Lab. of Endocrine and Metabolic Research, Milan, Italy; 2 Department of Clinical Sciences and Community Health, University of Milan, Milan, Italy; 3 Division of Biology and Biological Engineering, California Institute of Technology, Pasadena, CA USA; 4 Department of Neurobiology, The George S. Wise Faculty of Life Sciences and Sagol School of Neuroscience, Tel-Aviv University, Tel-Aviv, Israel

Correction to: *Scientific Reports* 10.1038/s41598-020-64077-2, published online 06 May 2020

The original version of this Article contained an incorrect title where, “Prokineticin receptor 2 affects GnRH3 neuron ontogeny but not fertility in zebrafish” was incorrectly given as “Knocking-down of the Prokineticin receptor 2 affects reveals its complex role in the regulation of the hypothalamus-pituitary-gonadal axis in the zebrafish model”.

This has now been corrected in the PDF and HTML versions of the Article.

