# Supplementary material for: Knocking-down of the Prokineticin receptor 2 affects reveals its complex role in the regulation of the hypothalamus-pituitary-gonadal axis in the zebrafish model
Source: Sci Rep. 2020 May 6;10:7632. doi: 10.1038/s41598-020-64077-2 (PMC7203128; doi:10.1038/s41598-020-64077-2)
Supplement: Supplementary file 1 — Supplementary information. [file 41598_2020_64077_MOESM1_ESM.doc]

**Prokineticin receptor 2 affects GnRH3 neuron ontogeny but not fertility in zebrafish**

Ivan Bassi^1^, Francesca Luzzani^1^, Federica Marelli^1,2^, Valeria Vezzoli^1^, Ludovica Cotellessa^1,2^, David A. Prober^3^, Luca Persani^1,2^, Yoav Gothilf^4^, Marco Bonomi^1,2,*^

^1^IRCCS Istituto Auxologico Italiano, Division of Endocrine and Metabolic Diseases & Lab. of Endocrine and Metabolic Research, Milan, Italy;

^2^Department of Clinical Sciences and Community Health, University of Milan, Milan, Italy

^3^Division of Biology and Biological Engineering, California Institute of Technology, Pasadena, CA, USA.

^4^Department of Neurobiology, The George S. Wise Faculty of Life Sciences and Sagol School of Neuroscience, Tel-Aviv University, Tel-Aviv, Israel*.*

Correspondence:

Marco Bonomi, MD

Dept. of Clinical Sciences & Community Health, University of Milan

IRCCS Istituto Auxologico Italiano, Division of Endocrine and Metabolic Diseases

Ospedale San Luca, Piazzale Brescia 20 - 20149 Milan, Italy

Phone: +39 02619112390

Fax: +39 02619113033

Email: [m.bonomi@auxologico.it;](mailto:m.bonomi@auxologico.it;) marco.bonomi@unimi.it

**Supplementary materials**

**Table S1.** Real-Time PCR primers used:

| *prokr1a* FW | | 5’ TTGTGCTTTGTGTCTCCATTAAC 3’ |
| --- | --- | --- |
| *prokr1a* REV | | 5’ TTATCCAGACCCCAAATATAAGC 3’ |
| *prokr1b* FW | | 5’ AGGTTTTGTGCGCCTCAGTTAAT 3’ |
| *prokr1b* REV | | 5’ CGACCCACACTCCTGTGATCAAA 3’ |
| *eef1a* FW | 5’ CTGGTGTCCTCAAGCCTGGTA 3’ | |
| *eef1a* REV | 5’ ACTTGACCTCAGTGGTTACATTGG 3’ | |
| *lhβ* FW^1^ | 5` ATAGTGGTTTGTTCTGGAGAC 3` | |
| *lhβ* REV^1^ | 5` GATGTACAGCTGAAGTCACAG 3` | |
| *fshβ* FW^1^ | 5` CAGATGAGGATGCGTGTGC 3` | |
| *fshβ* REV^1^ | 5` ACCCCTGCAGGACAGCC 3` | |
| *gnrh3* FW^2^ | 5` TGGAGGCAACATTCAGGATGT 3` | |
| *gnrh3* REV^2^ | 5` CCACCTCATTCACTATGTGTATTGG 3` | |

**Table S2.** In situ primers used

| *prokr1a* FW | 5’GGTACATGGCTATCGTTCACC 3’ |
| --- | --- |
| *prokr1a* REV | 5’CCTAACGCTCACAAAGCACA 3’ |
| *prokr1b* FW | 5’ATATGGCCATCGTTCATCCT 3’ |
| *prokr1b* REV | 5’CAGAACGATCCGCTTGAAGT 3’ |
| *GnRH3* FW | 5’ AGCATGGAGTGGAAAGGAAG 3’ |
| *GnRH3* REV | 5’ AGCCCATCTGTTCCTTCAGT 3’ |

**Table S3**. Morpholino`s sequences:

| *prokr1a-MO* | 5’ATTTAAGACAAGCACTCACCTGTCC 3’ |
| --- | --- |
| *prokr1b-MO* | 5’TATACCTGAACACAGAGACCACAGT3’ |

**Table S4**. Genotyping primers :

| *prokr1bGen FW*^3^ | 5’ TGAGCGTAATGCTAATGGTCT 3’ |
| --- | --- |
| *prokr1bGen REV*^3^ | 5’ CCAGAGTGGCGATAAACACA 3’’ |

1. Liu, Y. *et al.* Genetic evidence for multifactorial control of the reproductive axis in zebrafish. *Endocrinology* (2017) doi:10.1210/en.2016-1540.

2. Spicer, O. S. *et al.* The gonadotropin-inhibitory hormone (Lpxrfa) system’s regulation of reproduction in the brain-pituitary axis of the zebrafish (Danio rerio)t. *Biol. Reprod.* (2017) doi:10.1093/biolre/iox032.

3. Chen, S. *et al.* Light-Dependent Regulation of Sleep and Wake States by Prokineticin 2 in Zebrafish. *Neuron* **95**, 153-168.e6 (2017).

**Figure S1**

**
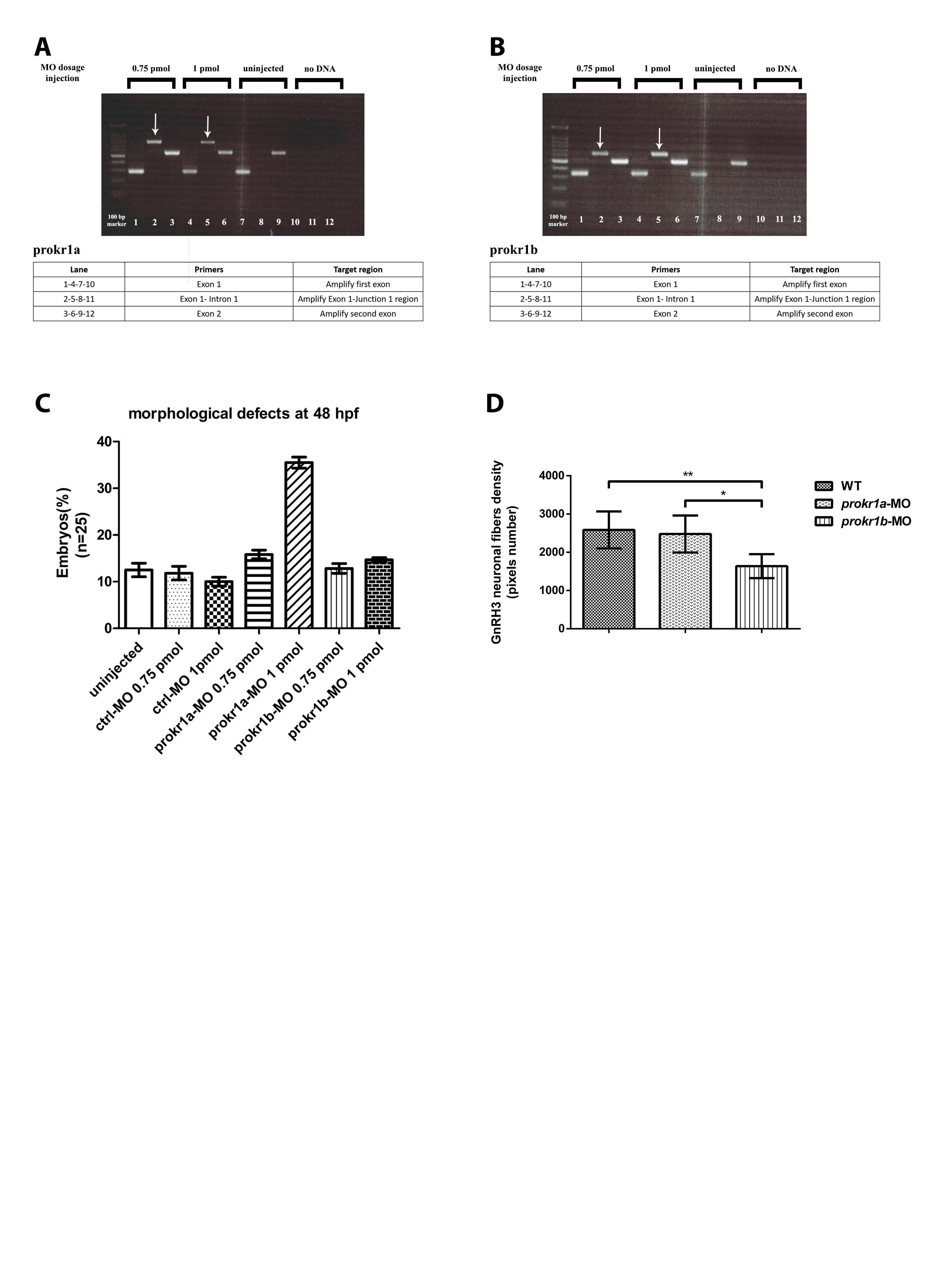
**

**Figure S1.** PCR conducted on cDNA extracted from 24 hpf embryos that had been injected with *prokr1a*- and *prokr1b*-specific MOs shows that both MOs cause an intron retention (A and B white arrows). Analysis of morphological defects suggested that 1 pmol of the *prokr1a* MO is toxic, while the *prokr1b* MO does not cause malformations at the concentrations tested (C). Quantification of GnRH3 fiber network density at 48hpf confirmed that prokr1a-MO is not affecting the migration of GnRH3 neurons.

**Figure S2**

**
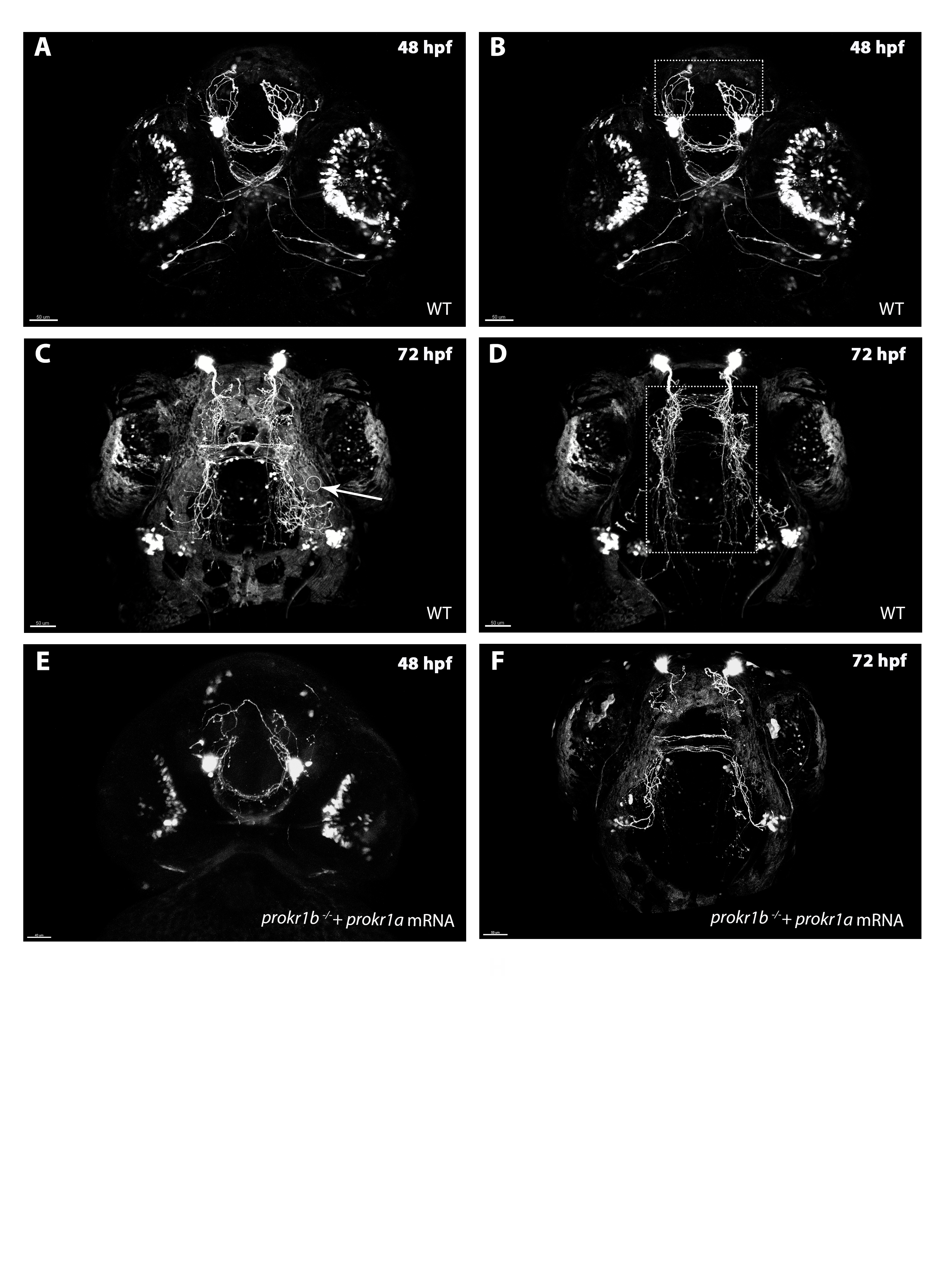
**

**Figure S2.** Panels B and D show the regions of interest (ROI, dashed boxes) used for GnRH3 fiber quantification in 48 hpf and 72 hpf embryos. To prevent background fluorescence from affecting the measurements at 72 hpf we subtracted to each image the correspondently background (C circle pointed by the arrow). Panel E and F shown that injection of *prokr1a* mRNA is not able to rescue the normal architecture of GnRH3 fibers observed in A and C.
